# Huaier Extract Attenuates Acute Kidney Injury to Chronic Kidney Disease Transition by Inhibiting Endoplasmic Reticulum Stress and Apoptosis via miR-1271 Upregulation

**DOI:** 10.1155/2020/9029868

**Published:** 2020-12-10

**Authors:** Jing-Ying Zhao, Yu-Bin Wu

**Affiliations:** Department of Pediatrics, Shengjing Hospital of China Medical University, Shenyang 110004, China

## Abstract

Endoplasmic reticulum stress (ERS) is strongly associated with acute kidney injury (AKI) to chronic kidney disease (CKD) transition. Huaier extract (HE) protects against kidney injury; albeit, the underlying mechanism is unknown. We hypothesized that HE reduces kidney injury by inhibiting ERS. In this study, using an AKI-CKD mouse model of ischemia-reperfusion injury (IRI), we evaluated the effect of HE on AKI-CKD transition. We also explored the underlying molecular mechanisms in this animal model and in the HK-2 human kidney cell line. The results showed that HE treatment improved the renal function, demonstrated by a significant decrease in serum creatinine levels after IRI. HE appreciably reduced the degree of kidney injury and fibrosis and restored the expression of the microRNA miR-1271 after IRI. Furthermore, HE reduced the expression of ERS markers glucose-regulated protein 78 (GRP78) and C/EBP homologous protein (CHOP) and inhibited apoptosis in the IRI group. This in vivo effect was supported by in vitro results in which HE inhibited apoptosis and decreased the expression of CHOP and GRP78 induced by ERS. We demonstrated that CHOP is a target of miR-1271. In conclusion, HE reduces kidney injury, probably by inhibiting apoptosis and decreasing the expression of GRP78 and CHOP via miR-1271 upregulation.

## 1. Introduction

The incidence of acute kidney injury (AKI) and chronic kidney disease (CKD) is increasing in recent years. Studies have shown that AKI is an independent risk factor for CKD [1]. AKI is associated with maladaptive repair, leading to fibrosis and CKD. The development of CKD from AKI is known as AKI-CKD transition [1, 2]. The risk of AKI-CKD transition can reach 25.8%, and the risk of developing end-stage renal failure from AKI is 8.6% [1] Therefore, preventing AKI-CKD transition is crucial for reducing the social and economic burden of CKD.

Studies have shown that after AKI, even in cases in which serum creatinine levels return to baseline, cellular inflammatory responses in the kidney activate molecular pathways, leading to fibrosis, long-term renal insufficiency due to nephron loss, tubular cell damage, maladaptive repair, interstitial fibrosis (IF), hypoxic endothelial dysfunction, epigenetic changes, and epithelial cell cycle arrest. These changes contribute to AKI-CKD transition [1, 3–6]. Under these pathological conditions, hypoxia, nutrient deficiency, ATP depletion, reactive oxygen species production, and impaired calcium homeostasis induce endoplasmic reticulum (ER) stress. The ER is a multifunctional eukaryotic organelle and the main site of protein synthesis, modification, and transport. Several factors can potentially cause the abnormal accumulation of unfolded and misfolded proteins in the ER, resulting in ER stress (ERS). The role of ERS in kidney injury has become a hot topic in recent years [7–9]. ERS participates in epithelial-mesenchymal transition (EMT) in proximal renal tubules, and EMT is a major contributor to renal fibrosis (RF). Thapsigargin (TG) increases the expression of C/EBP homologous protein (CHOP) and glucose-regulated protein 78 (GRP78) in the HK-2 human kidney cell line in vitro, triggering ERS and EMT responses, including the upregulation of *α*-smooth muscle actin (*α*-SMA), a marker of RF, and downregulation of E-cadherin [10, 11]. Moreover, persistent ERS can induce apoptosis [12]. An in vivo study demonstrated that ERS occurred in proximal tubules during AKI-CKD transition in an IRI model [13]. Two to 14 days after reperfusion, the levels of p-PERK, GRP78, and CHOP increased, and the inhibition of ERS by 4-PBA and TUDCA prevented AKI-CKD transition by reducing renal tubular cell apoptosis, inflammation, autophagy, atrophy, and fibrosis. Therefore, drugs that selectively inhibit ERS can potentially delay AKI-CKD transition.

Huaier, in Chinese pinyin, is a light-brown toadstool fungus that grows on the trunk of acacia trees. This fungus has been used in Chinese medicine for approximately 1500 years for treating multiple diseases. Recent studies show that the active ingredient in Huaier extract (HE) is a proteoglycan composed of 41.5% polysaccharides, 12.93% amino acids, and 8.72% water. The structure of this proteoglycan is similar to that of other immunomodulatory chemicals isolated from other Basidiomycota fungi. HE modulates innate immunity by stimulating cytokine release and producing reactive oxygen species and nitric oxide. Potential side effects are nausea and emesis [14]. Studies have shown that HE protects against kidney injury. However, the role of HE in AKI-CKD transition and ERS is incompletely understood. We hypothesize that HE protects renal tubular epithelial cells from apoptosis and reduces renal IF by inhibiting ERS. This hypothesis was tested using an IRI mouse model and a TG-induced cell model of AKI-CKD transition. This study assesses the role of HE in AKI-CKD transition and its mechanism of action.

## 2. Materials and Methods

### 2.1. Experimental Animals and In Vivo Model

Seventy-two male C57BL/6 mice (Changsheng Biological Co., Ltd., Liaoning Province) aged 6-8 weeks were randomly divided into three groups: sham group, AKI-CKD group (IRI), and IRI group treated with HE (IRI + HE), with 24 mice in each group. The IRI group was further divided into four subgroups according to the time elapsed after IRI: 24 h, and 3, 7, and 28 days, with six mice in each subgroup. The animals were housed in the specific-pathogen-free laboratory of the Experimental Center of Shengjing Hospital affiliated to the China Medical University.

The mice were allowed to adapt to the new environment for 1 week. Mice were anesthetized by intraperitoneal injection of 5 mg/mL sodium pentobarbital at a dose of 50 mg/kg body weight. In the IRI group, the blood vessels of the left renal pedicle were clamped for 35 min (at 38°C) under anesthesia, and reperfusion was performed. The sham group received all surgical procedures except for blockage of blood flow. The IRI + HE group received HE (Qidong Gaitian Pharmaceutical Co., Ltd., China) by oral gavage at a daily dose of 6 g/kg from days 3 to 28 after IRI to exclude the potential effect of HE on initial AKI.

### 2.2. Cell Culture

HK-2 cells were purchased from the Chinese Type Culture Collection and cultured in DMEM high glucose medium (Hyclone, USA) supplemented with 10% fetal bovine serum (Hyclone, USA) in a humidified atmosphere of 5% CO_2_ at 37°C. The cells were divided into three groups: (1) normally growing HK-2 cells (control), (2) TG group, which was treated with 200 nmol/L TG (Sigma) for 24 h, and (3) HE-treated group (TG + HE), which was treated with 100 *μ*g/mL HE for 24 h and cotreated with 200 nmol/L TG for 24 h.

### 2.3. Renal Function Test

Blood was collected from the tail vein of mice at 24 h, and 3, 7, and 28 days after IRI and was kept at room temperature (RT) for 2 h. Coagulated blood was centrifuged at 4°C for 10 min. Serum was collected and stored at –20°C for further use. Blood creatinine levels were measured enzymatically using an automatic biochemical analyzer (model ci16200, Abbott Laboratories).

### 2.4. HE and Masson Trichrome Staining

Mice were sacrificed 28 days after modeling. Kidneys were removed by laparotomy, fixed with 10% paraformaldehyde, and embedded in paraffin. Paraffin sections were stained with hematoxylin-eosin and Masson's trichrome. Morphological changes in kidney tissues, collagen formation, and the degree of RF were determined by light microscopy.

### 2.5. Immunohistochemistry

Paraffin sections were heated at 60°C for 120 min, deparaffinized, rehydrated, and subjected to antigen retrieval. The sections were incubated with primary antibodies against GRP78 (1 : 100) and CHOP (1 : 200) at 4°C overnight, washed with PBS, and incubated with HRP-labeled secondary antibody for 40 min at RT. The samples were stained with DAB and hematoxylin, dehydrated, mounted on glass slides, and imaged under a light microscope.

### 2.6. Western Blotting

The total protein of kidneys and HK-2 cells was extracted using a protein extraction kit (OriGene Technologies, Inc.; Beijing, China) and quantitated using the BCA method. Proteins were analyzed by SDS-PAGE and transferred to a PVDF membrane. The membrane was blocked with skim milk for 2 h at RT and incubated with primary antibodies against GRP78, CHOP, and GAPDH (Abcam, Cambridge, UK). The samples were washed with PBS and incubated with horseradish peroxidase-labeled goat anti-rabbit-IgG (1 : 3000) (SE134; Solarbio, Beijing, China) for 2 h at 37°C. Protein bands were visualized by enhanced chemiluminescence using an Azure C300 imaging system (Azure Biosystems, Dublin, CA, USA) and quantified using the system software. The relative expression was calculated using GAPDH as the internal control.

### 2.7. Real-Time PCR

The total RNA of each sample was purified using a total RNA extraction kit (Takara, Japan). cDNA was reverse transcribed from total RNA and amplified and quantitated by real-time polymerase chain reaction. The following primer sequences were used: miRNA-1271-5p: forward, 5′-CAGCACTTGGCACCTAGCA-3′; reverse, 5′-TATGGTTGTTCTCCTCTCTGTCTC-3′; U6: forward, 5′-CGCAAGGATGACACGCAAAT-3′; reverse, 5′-GCAGGGTCCGAGGTATTC-3′; CHOP: forward, 5′-TCCTGCGTCGGTGTATTC-3′; reverse, 5′-CGTGAGTTGGTTCTTGGC-3′; GRP78: forward, 5′-GGAGCAGGAGAATGAGAG-3′; reverse, 5′-GACAGACAGGAGGTGATG-3′; GAPDH: forward, 5′-TGTTCCTACCCCCAATGTGTCCGTC-3′; reverse, 5′-CTGGTCCTCAGTGTAGCCCAAGATG-3′. Data were expressed as Ct values. Experiments were performed in triplicate. The relative expression levels were calculated using the 2^–*ΔΔ*Ct^ method and normalized to GAPDH levels.

### 2.8. TUNEL Assay

Each tissue section was incubated with 50 *μ*L of 0.1% Triton X-100 in 0.1% sodium citrate for 8 min at RT and rinsed with PBS three times for 5 min each. The sections were incubated with a reaction mixture containing an enzyme solution and a label solution (1 : 9, v:v) at 37°C for 60 min in a humidified incubator in the dark. The sections were rinsed with PBS, counterstained with DAPI for 5 min in the dark, rinsed with PBS, mounted with Vectashield mounting medium, and examined under a light microscope.

### 2.9. Analysis of Cell Apoptosis by Flow Cytometry

HK-2 cells were seeded on T25 culture flasks at a density of 5 × 10^5^ cells per flask and were cultured in a humidified atmosphere of 5% CO_2_ at 37°C. Cells were harvested after 24 hours of culture and washed twice with PBS by centrifugation at 800 rpm for 5 min. The pellet was resuspended in 500 *μ*L of binding buffer and incubated with 5 *μ*L of FITC-conjugated annexin V and 5 *μ*L of propidium iodide for 15 min at RT in the dark.

### 2.10. Luciferase Assay

The 3′ UTR region of CHOP was cloned into the pMIR-reporter luciferase vector. A triple mutation at the predicted microRNA (miRNA) binding site in this region was made as a control. The miRNA mimic was cotransfected with the plasmid containing the wild type or mutant CHOP 3′ UTR into 293 T cells. The firefly luciferase activity was normalized to the Renilla luciferase activity. Assays were performed in triplicate. The sequence of the wild-type and mutant CHOP 3′ UTR was 5′-ACAATTGGGAGCATCAGTCCCCCACTTGGGCCACACTACCCACCTTTCCCAGAAGTGGCTACTGACTACCCTCTCACTA**GTGCCAA**TGATGTGACCCTCAATCCCACATACGCAGGGGGAAGGCTTGGAGTAGACAAAAGGAAAGGTCTCAGCTTGTATATAGAGATTGTACATTTATTTATTACTGTCCCTATCTATTAAAGTGACTTTCTATGAGCC-3′ and 5′-ACAATTGGGAGCATCAGTCCCCCACTTGGGCCACACTACCCACCTTTCCCAGAAGTGGCTACTGACTACCCTCTCACTA**CACGGTT**TGATGTGACCCTCAATCCCACATACGCAGGGGGAAGGCTTGGAGTAGACAAAAGGAAAGGTCTCAGCTTGTATATAGAGATTGTACATTTATTTATTACTGTCCCTATCTATTAAAGTGACTTTCTATGAGCC-3′, respectively. The mutated bases are shown in bold.

### 2.11. Statistical Analysis

Statistical analysis was performed using SPSS version 21.0. Data were expressed as means and standard deviations. The normality of the distribution and variance homogeneity was tested using two-tailed unpaired Student's *t*-test. The groups were compared using one-way analysis of variance, and a *P* value of less than 0.05 indicated a statistically significant difference.

## 3. Results

### 3.1. HE Reduced Renal Damage and Fibrosis and Upregulated miR-1271 during AKI-CKD Transition

**Figure 1:**
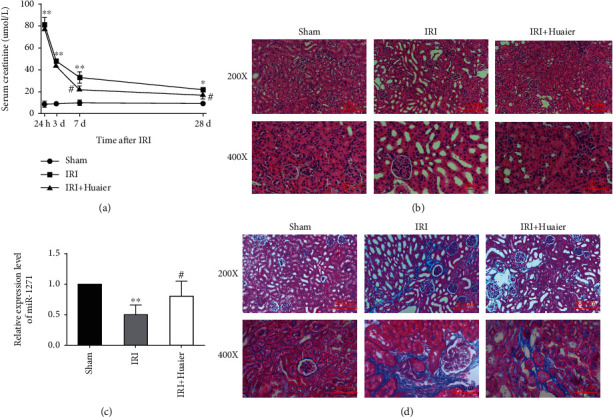
Huaier extract reduces renal damage and fibrosis and upregulates miR-1271 during AKI-CKD transition. (a) *S*erum creatinine level over time. (b) Photomicrographs of kidney sections 28 days after ischemia-reperfusion injury (IRI) (hematoxylin-eosin staining). Scale bars: 100 *μ*m (×200) or 50 *μ*m (×400). (c) Relative expression of miR-1271 by real-time PCR. (d) Photomicrographs of kidney sections showing collagen deposition 28 days after IRI (Masson trichrome staining). ^∗^*P* < 0.05; ^∗∗^*P* < 0.01 (vs. the sham group); ^#^*P* < 0.05; ^##^*P* < 0.01 (vs. the IRI group).

The serum creatinine level increased approximately 10-fold at 24 h after IRI when compared with the sham group. This level decreased over time in the IRI and IRI + HE groups but was higher than that of the sham group during the 28-day study period. HE treatment in the IRI + HE group started on day 3 after IRI, and the creatinine levels in this group were significantly lower than those in the IRI group on days 7 and 28 (Figure 1(a)). IRI decreased the expression of miR-1271, and HE abrogated this effect (Figure 1(c)). Renal histology is shown in Figures 1(b) and 1(d). In the sham group, the tubular basement membrane was intact, the tubular epithelial cell structure was clear, and there was no inflammatory cell infiltration. In the IRI group, renal tubules were expanded, and tubular epithelial cells were swollen, degenerated, necrotic, and partially atrophied, with IF. HE treatment reduced the degree of IF and prevented tubular epithelial cell atrophy, compensatory hypertrophy, and interstitial edema (Figure 1(b)). In the sham group, the collagen fibers around the tubules were weakly stained. In the IRI group, the tubular interstitium was damaged, and the renal interstitium was widened, with collagen deposition and multifocal fibrosis. Furthermore, the number of interstitial cells and amount of extracellular matrix increased. HE treatment partially decreased collagen deposition (Figure 1(d)).

### 3.2. HE Downregulates CHOP and GRP78 In Vivo

**Figure 2:**
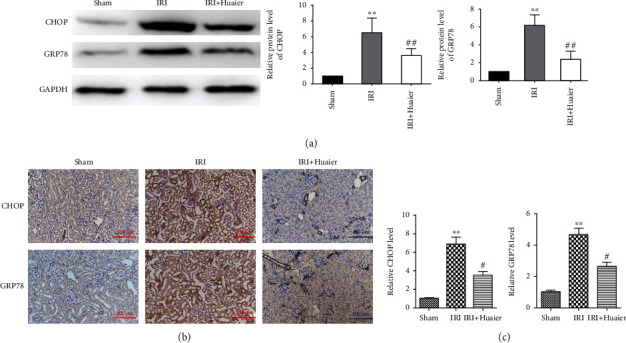
Huaier extract downregulates the CHOP and GRP78 protein expression in vivo. (a) Western blot analysis of the expression of GRP78 and CHOP in kidney sections 28 days after ischemia-reperfusion injury (IRI). (b) Immunohistochemical analysis of the expression of GRP78 and CHOP 28 days after IRI. Scale bar : 100 *μ*m. (c) The mRNA expression of GRP78 and CHOP by real-time PCR. ^∗∗^*P* < 0.01 (^∗^vs. the sham group); ^#^*P* < 0.05; ^##^*P* < 0.01 (vs. the IRI group).

The mRNA and protein levels of ERS markers GRP78 and CHOP in the kidney were measured by Western blotting, immunofluorescence, and real-time PCR to evaluate ERS in AKI-CKD transition and the effect of HE on ERS. IRI increased the protein levels of GRP78 and CHOP, and HE reduced this effect (Figure 2(a)). Immunofluorescence staining revealed that IRI increased the GRP78 and CHOP protein expression, whereas HE treatment restored protein levels to some extent (Figure 2(b)). Furthermore, IRI increased the mRNA expression of GRP78 more than 4-fold and CHOP more than 6-fold, and HE attenuated this effect (Figure 2(c)), demonstrating that HE treatment prevented ERS during AKI-CKD transition.

### 3.3. HE Downregulates CHOP and GRP78 and Upregulates miR-1271 in HK-2 Cells

**Figure 3:**
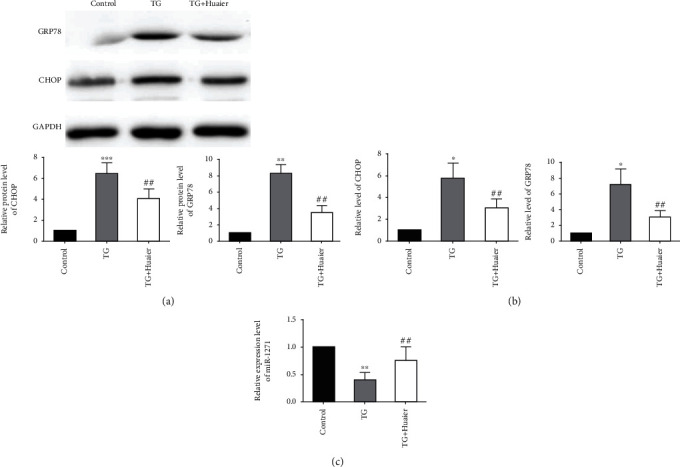
Huaier extract downregulates CHOP and GRP78 and upregulates miR-1271 in an endoplasmic reticulum stress model in HK-2 human kidney epithelial cells. (a) Western blot analysis of the expression of GRP78 and CHOP in kidney sections. (b) The mRNA expression of GRP78 and CHOP by real-time PCR. (c) The relative expression of miR-1271 by real-time PCR. ^∗∗^*P* < 0.01, (^∗^vs. the sham group),^#^*P* < 0.05, ^##^*P* < 0.01 (vs. the ischemia-reperfusion injury group). ^∗^*P* < 0.05 (vs. the control group), ^∗∗^*P* < 0.01, ^##^*P* < 0.01 (vs. the thapsigargin-treated group).

The effect of HE on ERS was evaluated in HK-2 human kidney epithelial cells to confirm the in vivo findings. ERS was induced with TG. TG increased the protein expression of CHOP and GRP78, indicating that ERS was successfully established in this cell line, and HE treatment abrogated this effect (Figure 3(a)). Similarly, TG dramatically increased the mRNA expression of GRP78 and CHOP, and HE reduced this effect (Figure 3(b)). Conversely, TG significantly decreased the expression of miR-1271, and HE prevented this effect (Figure 3(c)), demonstrating that HE could potentially reduce ERS and cell damage in HK-2 cells.

### 3.4. miR-1271 Binds to the 3′ UTR of CHOP mRNA

**Figure 4:**
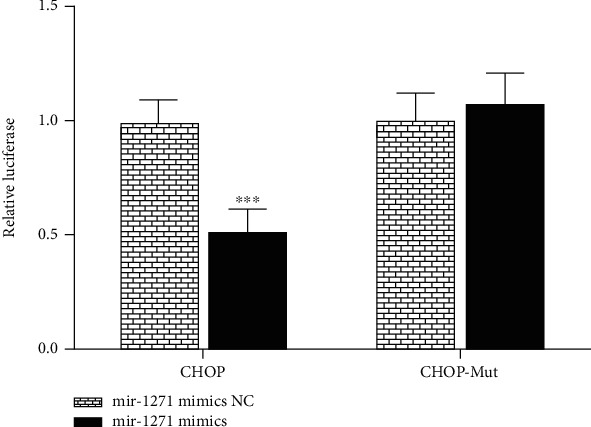
miR-1271 binds to the 3′ untranslated region of CHOP mRNA.

The 3′ UTR region of *CHOP* was cloned into the pMIR-reporter luciferase vector to evaluate whether miR-1271 targets ERS-related proteins. The miR-1271 mimic significantly downregulated the CHOP 3′ UTR luciferase activity compared with the negative control, and mutations at the predicted miR-1271 binding site abolished this effect (Figure 4). These results suggest that HE reduces ERS-induced renal damage by inhibiting the CHOP expression via miR-1271 upregulation.

### 3.5. HE Reduces Apoptosis

**Figure 5:**
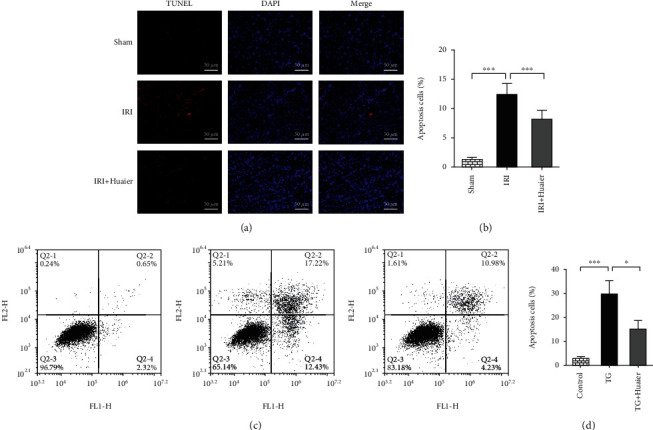
HE inhibits apoptosis. (a) The percentage of apoptotic cells was determined using the TUNEL assay (×400 magnification). (b) Percentage of apoptotic cells in the study groups. (c) Detection of FITC-conjugated annexin V and propidium iodide in apoptotic cells by flow cytometry. (d) Percentage of apoptotic cells in the study groups (^∗^*P* < 0.05; ^∗∗^*P* < 0.01; ^∗∗∗^*P* < 0.001).

Apoptosis was measured in tissues and HK-2 cells using the TUNEL assay (Figures 5(a) and 5(b)) and flow cytometry (Figures 5(c) and 5(d)). Annexin V/PI double staining showed that IRI drastically increased the number of apoptotic cells, and HE attenuated this effect (Figure 5(b)). Flow cytometry showed that TG increased the percentage of apoptotic cells and HE reduced this effect (Figures 5(c) and 5(d)).

## 4. Discussion

HE has been shown to protect against renal injury. However, the mechanism of action of HE is unknown. Since ERS plays an important role in the pathogenesis of AKI-CKD transition, we hypothesized that the protective effect of HE is mediated by the inhibition of ERS. Our results demonstrated that HE treatment improved IRI-induced renal injury, decreased RF, restored the expression of miR-1271 after IRI, and reduced ERS by decreasing the expression of GRP78 and CHOP. This result was supported by in vitro experiments in the HK-2 human kidney cell line. HE attenuated the TG-induced increase in the CHOP and GRP78 expression. Furthermore, CHOP was a target gene for miR-1271. Therefore, HE prevents kidney injury, possibly by inhibiting the expression of GRP78 and CHOP through the upregulation of miR-1271.

Maladaptive repair after AKI permanently damages the kidney structure and function [15] and promotes RF [16]. Inflammation persists after AKI, even in cases of fast recovery of the kidney function, and leads to fibrosis. The risk of CKD is significantly higher in individuals with AKI than in those without AKI. CKD develops over many years. Although cytokines and other factors are involved in AKI-CKD transition, RF is a hallmark of CKD. Therefore, preventing RF after AKI is fundamental for reducing the social and economic burden of CKD.

HE ameliorated proteinuria and IgA deposition in the glomerular mesangium in patients with adriamycin and IgA nephropathy [17–20] and protected mice against cisplatin-induced AKI [21]. HE inhibited EMT and RF in a unilateral ureteral obstruction (UUO) renal IF mouse model [22]. However, no studies have examined the role and mechanism of action of HE in AKI-CKD transition. Our results showed that HE prevented AKI-CKD transition, improved RF, and reduced the degree of renal damage and fibrosis using an IRI mouse model.

Moderate ERS can trigger a protective response in mammalian eukaryotic cells, restore ER homeostasis, and improve cell survival. However, excessive ERS triggers apoptosis, which further aggravates tissue damage. Xu et al. [23] showed that ERS promoted RF, whereas SIRT1 reduced RF by inhibiting ERS in a UUO IF model [24]. Moreover, ERS inhibitors suppressed the *α*-SMA expression [25], suggesting that ERS inhibition can potentially prevent RF and delay AKI-CKD transition.

The increased expression of GRP78 in the ER lumen is an indicator of ERS [26]. Unfolded proteins accumulate in the ER during ERS, causing GRP78 to separate from its sensor proteins, triggering the activation of downstream signaling pathways and protein refolding [27]. CHOP is mainly expressed in the cell nucleus, and its expression is very low under normal physiological conditions. ERS upregulates CHOP, which controls the expression of downstream apoptosis-related genes and mediates apoptosis in ERS. Long-term excessive ERS causes cell toxicity [28, 29] and induces apoptosis through multiple cytokines. Lempiainen et al. [30] found that CHOP knockout significantly decreased the degree of kidney damage in IRI mice compared with wild-type mice, indicating that CHOP is implicated in kidney damage.

In this study, IRI significantly increased the CHOP and GRP78 expression, suggesting that ERS is involved in renal injury during AKI-CKD transition, and this result is consistent with a previous study [13]. Furthermore, HE downregulated GRP78 and CHOP in the renal tissue, thereby reducing the degree of ERS after IRI. TG is a calcium-ATPase inhibitor that induces calcium release from the ER into the cytoplasm, triggering ERS [9]. Treatment of HK-2 cells with TG for 24 h induced ERS and EMT, simulating AKI-CKD transition [7]. In this study, ERS was induced in HK-2 cells using TG. It has been reported that Huaiqihuang, which contains mostly HE, reduces ERS by decreasing the podocyte GRP78 and CHOP expression and protects mice against high glucose-induced podocyte damage [31] and cisplatin-induced nephrotoxicity by inhibiting the apoptosis of renal tubular epithelial cells [21]. Furthermore, HE treatment significantly decreased apoptosis and, consequently, protected renal tubular cells after IRI and TG-induced ERS under our experimental conditions. Fanget al. [32] found that HE improved the kidney function by reducing cisplatin-induced kidney damage and apoptosis, which is consistent with our findings. MiRNAs are small regulatory noncoding RNAs that bind to the mRNA 3′ UTR region of target genes and regulate gene expression at the posttranscriptional level by inhibiting mRNA translation or facilitating mRNA degradation. The Target Scan bioinformatics website predicted that miR-1271-5p targeted CHOP mRNA and inhibited EMT in tumors [33], suggesting that miR-1271 suppressed RF by inhibiting the CHOP expression and EMT. It has been reported that Huaiqihuang can inhibit cancer progression by regulating miRNAs [34]. Therefore, HE may attenuate AKI-CKD transition by inhibiting the CHOP pathway in ERS via miR-1271 upregulation. We demonstrated that HE treatment prevented the downregulation of miR-1271 after AKI. This result was supported by an in vitro experiment, in which the miR-1271 expression was decreased during AKI-CKD transition, and HE abrogated this effect. Furthermore, the results of dual-luciferase assays confirmed that miR-1271 binds to CHOP.

## 5. Conclusions

Our results show that HE reduces ERS-induced renal fibrosis and apoptosis during AKI-CKD transition by inhibiting CHOP through the upregulation of miR-1271. Notwithstanding, other mechanisms by which HE improves renal function need to be explored.

## Data Availability

The datasets used and/or analyzed during the current study are available from the corresponding author on reasonable request.
